# The relationship of severity in diastasis recti abdominis and pelvic floor dysfunction: a retrospective cohort study

**DOI:** 10.1186/s12905-021-01194-8

**Published:** 2021-02-15

**Authors:** Hui Fei, Yun Liu, Mengxiong Li, Juan He, Lixiang Liu, Juanhua Li, Ying Wan, Tian Li

**Affiliations:** 1 Pelvic Floor Disorders Center, The Seventh Affiliated Hospital, Sun Yat-Sen University, Shenzhen, GuangDong Province China; 2 Department of Gynecology and Obstetric, The Seventh Affiliated Hospital, Sun yet-san University, Shenzhen, GuangDong Province China

**Keywords:** Diastasis recti abdominis, Pelvic floor muscle strength, Pelvic organ prolapse, Postpartum, Rectus abdominis muscle strength

## Abstract

**Background:**

Diastasis of rectus abdominis (DRA) refers to a separation of the rectus abdominis from the linea alba. This study aimed to investigate the association with the severity of DRA for developing pelvic floor dysfunction among women during the first year postpartum.

**Methods:**

This is a retrospective cohort study which collected data from 229 postpartum women. DRA was defined as a separation of ≥ 20 mm at any point 4.5 cm above, at and 4.5 cm below the umbilicus. The data for analysis includes pelvic organ prolapse quantification (POP-Q), medical history of urinary incontinence (UI), the strength of rectus abdominis muscle and pelvic floor muscle. The differences in women with and without DRA were compared with independent samples t-test and Chi-square test.

**Results:**

Prevalence of DRA was 82.6% during the first postpartum year. Cesarean section and multiple parturitions are recognized as risk factors for DRA due to the odds ratio in our study were 3.48 (95% CI 1.42–8.56), 3.20 (95% CI 1.59–6.45) respectively. There was no difference in the occurrence of UI and pelvic organ prolapse (POP) comparing women with and without DRA, even changing the cut-off values (inter-rectus distance = 20 mm, 30 mm, 40 mm, 50 mm) for determining DRA. The women with weak rectus abdominis muscle and pelvic floor muscle have no statistical difference in two group.

**Conclusion:**

The relationship of the diastasis recti abdominis and pelvic floor dysfunction has no connection, even with the severity of inter-rectus distance increasing.

## Introduction

Diastasis of rectus abdominis (DRA) is a condition defined as a separation of the rectus abdominis from the linea alba [1, 2]. Numerous studies have described the prevalence of DRA was between 27 and 100% [1, 3, 4]in the middle and late of pregnancy respectively, 30–68% in the postpartum period [4–7]. DRA is common in pregnancy and postpartum women [3]. Due to the variety of hormone changes during pregnancy, the abdominal muscles stretch affect by relaxin, progesterone and estrogen [8]. Some investigations showed DRA leads to a series of complications, including abnormal condition, lumbopelvic pain and external defects which result in lower body satisfaction [7, 9]. Pelvic floor dysfunction (PFD) mainly includes pelvic organ prolapse (POP), urinary incontinence (UI), and sexual dysfunction [10]. As we know, the risk factors for POP and UI are involved in parity, advancing age, and obesity [11–14]. The main risk factors for diastasis of rectus abdominis are obesity, multiparity, fetal macrosomia, flaccid abdominal muscles and multiple pregnancies [8]. The causes of diastasis recti abdominis is unclear, but a general belief of not only diastasis recti abdominis but pelvic floor dysfunction could lead to weak connective tissue [15]. There is contradictory evidence about the association of the diastasis recti abdominis and pelvic floor dysfunction [16]. As is known to all that DRA is not a primary cause of trouble or pain, but it may contribute to the development of lumbar pain or pelvic floor dysfunction [7]. Based on a previous research, it was noted that UI, POP and fecal incontinence occurs more often in women who have DRA than in women without DRA [9]. There was an investigation which showed the incidence of DRA in a urogynecological patient and described DRA has a relationship with pelvic floor dysfunction [9]. Abdominal muscles and pelvic floor muscles strength (PFMs) play an essential role in pelvic, abdominal dynamics. Patiens with DRA are more likely to develop pelvic floor muscle weakness; As a result, they are also more likely to cause UI and POP [17]. But some study did not support the correlation between diastasis of rectus abdominis, pelvic organ prolapse and lower-back pain [7, 16, 18, 19]. Thus, it is a controversial whether the sever DRA, which means the wider inter-rectus distance, could lead to the higher incidence of UI and POP. The purpose of this study is to explore the association with the severity of DRA for developing pelvic floor dysfunction among women during the first year postpartum.

In view of this, a better understanding of the association with DRA for developing pelvic floor dysfunction, including the occurrence of UI, POP among women during the first year postpartum is essential.

## Methods

### Study type and data collection

This study is a retrospective cohort study. All data comes from the database in Pelvic Floor disorders Center in the Seventh Affiliated Hospital of Sun yet-san University in China. It collected 229 women in our hospital. The postpartum women of were invited to participant in the study between 4th March 2019 and 9th December 2019.

The medical records have two main parts. One is the basal and clinical data, including age, race, occupation, pre-pregnancy body mass index (BMI), predelivery BMI, postpartum BMI, height, weight gain during pregnancy, delivery times, weeks of gestation, newborn birth weight, type of delivery and academic degree (Here we defined the bachelor degree or above as the highest cademic degree). Another part was the professional data, including inter-rectus distance, waistline, the POP quantification (POP-Q), medical history of UI, the strength of rectus abdominis muscle and pelvic floor muscle, symptoms of lumbago or dorsalgia.

The postpartum women get two different schedules in the Pelvic Floor disorders Center, which may be on the same day or not. An experienced gynaecologist collected the medical records, including the basal and clinical data, the medical history of UI and symptoms of lumbago or dorsalgia. The same experienced gynaecologist also did the ultrasound for inter rectus distance in one room. A professional physiotherapist who was blinded to the data collected by the gynaecologist performed the assessment of POP-Q and measured the waistline, the strength of rectus abdominis muscle and pelvic floor muscle in another room. The data was written in different files. The postpartum women submitted the file to a nurse who belongs to an independent third-party after finishing the examinations.

All data mentioned above recorded by the paper. All of the clinical examinations were performed by the same gynaecologist and the same trained professional physiotherapist in the Pelvic Floor disorders Center.

### Inclusion and exclusion criteria

The inclusion criteria are as follows: (1) women over 18 years of age; (2) women who had the postnatal follow-up during the first year.

Women were excluded if they meet one of the following conditions: (1) undergoing a severe illness; (2) uncompleted inter-rectus distance (IRD) records; (3) have a history of abdominal or lower back surgery except for cesarean section; (4) have a history of pelvic floor dysfunction.

In total, data from 229 women were collected. 215 women met the inclusion criteria, while 2 women were excluded because of incomplete IRD records. 213 women were enrolled in the statistical analyses.

### Measurements

**Fig. 1:**
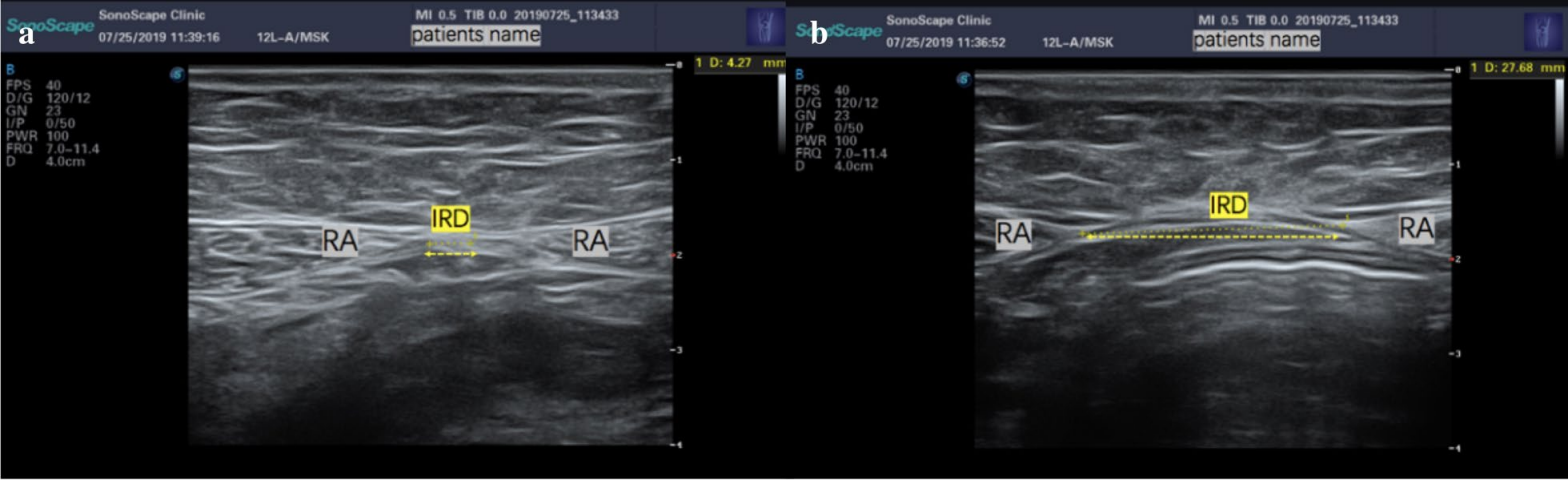
Ultrasound images. **a** Shows a normal situation for inter-rectus distance (< 20 mm); **b** Shows a situation of diastasis of rectus abdominis (≥ 20 mm); the subjects’ name are covered for safeguard subjects’ privacy. IRD, inter-rectus distance; RA, recti abdominis

Inter-rectus distance is measured using ultrasonic machine. The ultrasound equipment is the X5 (producted by SonoScape Medical Corp.), whose transducer footprint size is 60 mm x 18 mm and center frequency is 7.5 MHz. The participate was asked to lie on their back in a hook holding position (knees bent, feet flat on the table) with her arms on the table [20]. The measurement sites on the abdomen were marked 4.5 cm above, at and 4.5 cm below the umbilicus. DRA was considered as the distance between any point of rectus abdominis above the arcuate line is ≥ 20 mm (Fig. 1) [1, 21–23].

POP was assessed by the gynaecologists and the physiotherapist using Pelvic Organ Prolapse Quantification System (POP-Q) in lithotomy position. POP was considered as a POP-Q stage ≥ 2 for any parts, no POP as a POP-Q of 0 or 1 [6]. The situation of UI was assessed by the medical history. A history of uncontrolled urine leakage determined UI postpartum, which was defined as the automatic leakage of urine occuring ≥ 2 times/week, whether stress incontinence, urgency incontinence or mixed incontinence [6].

For measuring the strength of rectus abdominal muscles, we use the method of manual muscle test (MMT), which requires no equipment. Based on previous studies, all subjects were asked to lie down, bend their knees to 90° and put their feet on the ground. The strength evaluated the ability of the participant to lift the trunk [24, 25]. The examiner palpated the rectus abdominis muscles when participants were raising a trunk. It mainly consists of six scores from 0 to 5 defined in below:

- 0 No contraction
- 1 Flicker or trace contraction (examiners palpated the rectus abdominis muscles of subjects and let them raise the head or cough)
- 2 Raise the trunk with arms at two sides and only lift the head
- 3 Raise the trunk with arms outstretching above the plane of the body
- 4 Raise the trunk with arms crossing across the chest
- 5 Raise the trunk and claspe hands behind the head

For the pelvic floor muscles strength, We evaluate the pelvic floor muscle strength on two parts, i.e. type I and type II [26, 27] by manual muscle testing. The ability of how to contract the pelvic floor muscles (PFM) was trained by a professional physical therapist. The PFM strength for type I was decided by endurance, which is expressed as the length of time and power. The PFM strength for type II was assessed by a one-second maximal voluntary contraction and the time of the contraction after a one -minute rest. Subjects were trained to ‘contract-relax' as quickly and powerfully as possible in 6 s. The power was evaluated by the modified Oxford Scale, which is an optimized method for the determination of pelvic floor muscle strength and is widely used in clinical practice [28–30]. The contraction was a graded system:

- Level 0: No contractions of the pelvic floor muscles were felt in the examiner's fingers.
- Level 1: The examiner's fingers feel tremors or pulsations—very weak contractions.
- Level 2: The examiner's fingers felt weak contractions—an increase in muscle tension but no perceptible lifting or squeezing.
- Level 3: The examiner's fingers experienced moderate contractions – characterized by elevation of the posterior wall of the vagina and a feeling of compression at the base of the fingers (pectineal muscle) with adduction of the perineal body.
- Level 4: The examiner's fingers feel good contractions – which can counteract resistance and produce elevated posterior vaginal wall and perineal retraction. If two fingers (index and middle) are placed laterally or vertically into the vagina and separated, level 4 muscle contraction can crush them together against resistance.
- Level 5: The examiner's fingers felt powerful contractions—which can raise the back wall of the vagina against strong resistance and push the index and middle fingers together.

Besides, we classified the weakness group of rectus abdominal muscles and PFM strength as Level 0–1 [28–30].

### Statistical analysis

Statistical analyses were performed by IBM SPSS Statistics software, version 26. The statistical significance sets at *p* < 0.05. Categorical variables were described as a number or a percentage. Continuous variables were expressed as mean and standard deviation. The differences of the weight gain during pregnancy, education and parity used an independent sample t-test between women with DRA and without DRA if data were distributed normally. Due to the incidence of the pelvic floor dysfunction, just like the UI, prolapse and the percentage of cesarean section reported as categorical variables, their variations were analyzed using the Chi-square test. We calculated odds ratio (OR) for different risk factors reported as OR with 95% CI. Missing data were not replaced for estimation.

## Results

**Fig. 2:**
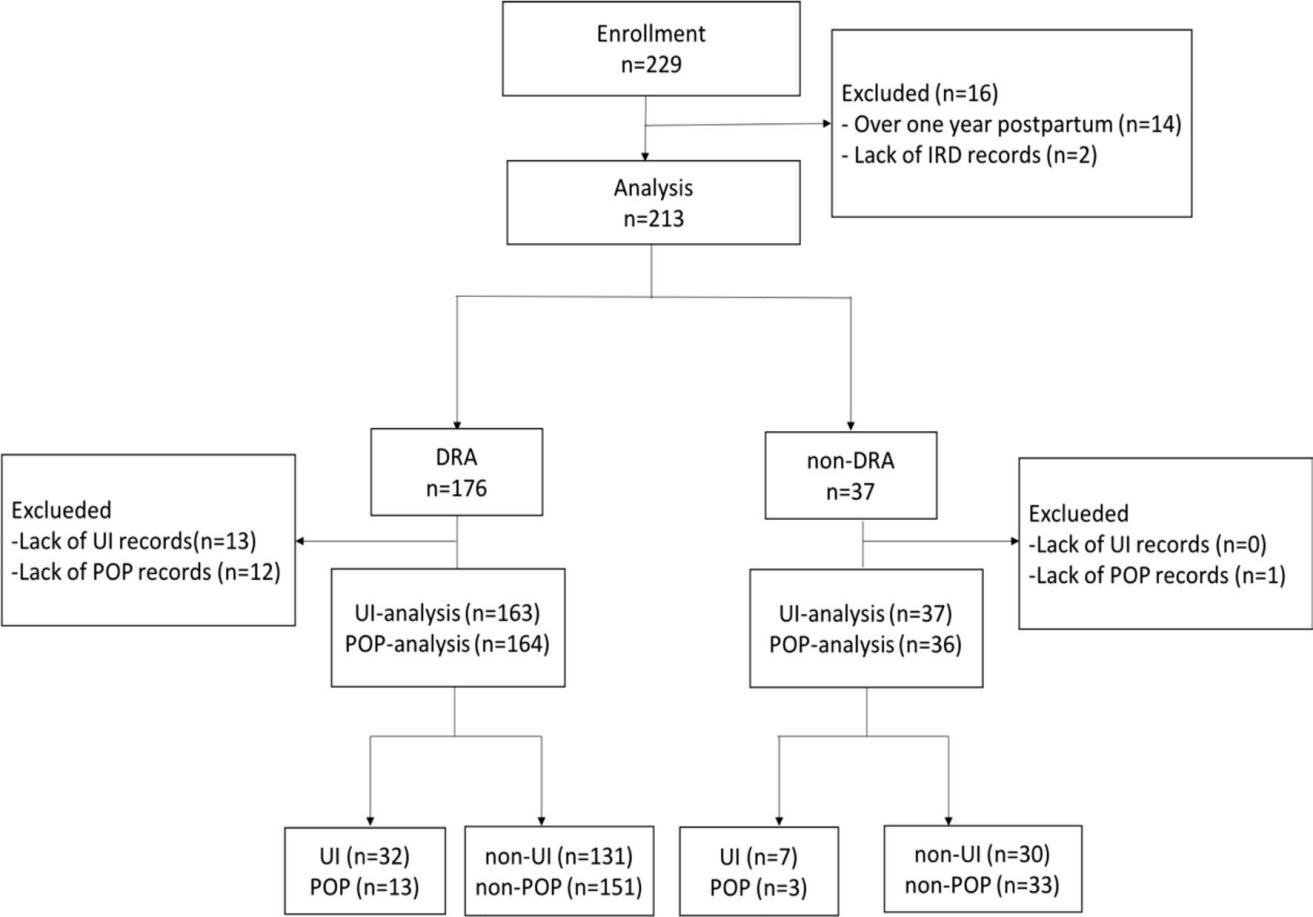
Flow chart. DRA, diastasis of rectus abdominis; IRD, inter-rectus distance; UI, urinary incontinence; POP, pelvic organ prolapses

There was a total of 229 adult women collected. Two hundred fifteen subjects were during the first year of postpartum. 2 subjects were excluded due to lack of the IRD records. Finally, 213 met the inclusion criteria and were analyzed (Fig. 2: Flow chart). The number of DRA and non-DRA was 176 (82.6%), 37 (17.4%) respectively. Basal data of two groups shown in Table 1. The average age and height in the two groups is different, and the women without DRA seemed like younger and higher. Even it showed the proportions of physical work and high education in a group of non-DRA were higher in the DRA group. They did not show statistical significance. Nevertheless, the history of cesarean section and multiple parturitions in DRA group was significantly higher than the non-DRA group as expected (39.8% vs 13.5%, *p* = 0.002; 56.3% vs 24.3%, *p* = 0.000).

**Table 1:** Basal and clinical data in group of DRA and Non-DRA

	DRA (n = 176)	Non-DRA (n = 37)	*p* value
Age (years)	30.60 ± 4.11 (21–40)	28.49 ± 3.40 (21–36)	0.004
Post-natal period (days)	66.65 ± 48.40 (30–365)	66.92 ± 57.43 (42–348)	0.888
*Occupation*			
Brainwork	141 (82.0%)	29 (78.4%)	0.610
Physical work	31 (18.0%)	8 (21.6%)	0.610
High education	131 (74.9%)	30 (81.1%)	0.421
Weight gain in pregnancy (kg)	13.55 ± 4.74 (3–27)	13.76 ± 4.03 (4.5–22)	0.799
Height (cm**)**	158.68 ± 5.10 (140–172)	161.23 ± 5.98 (153–176)	0.008
BMI pre-pregnancy (kg/m^2^)	20.83 ± 2.92 (16.23–28.67)	20.69 ± 2.55 (15.62–26.56)	0.781
BMI pre-delivery (kg/m^2^)	26.20 ± 3.39 (20.45–35.30)	26.01 ± 3.28 (19.44–33.59)	0.765
BMI post-delivery (kg/m^2^)	18.77 ± 9.12 (16.20–30.78)	17.28 ± 9.63 (17.53–26.06)	0.381
Cesarean section	70 (39.8%)	5 (13.5%)	0.002
Multiple parturitions	99 (56.3%)	9 (24.3%)	0.000

Occupation, high education, cesarean section, multiple parturitions showed as number of cases (valid percentage); Age, postnatal period, weight gain in pregnancy, height, BMI in different state showed as mean ± SD. BMI, body mass index

The incidence of UI and POP show in Table 2. In our study, there was no statistically significance in POP between two groups. The percentage of them in women with and without DRA was close (20.2% vs 18.9%, 7.9% vs 8.3%), and the occurrence of UI is more likely to happen in women with DRA. However, the results showed no statistically significant difference (*p* > 0.05). None of women in our study had a 0 grade in the Oxford grading. The women with weak rectus abdominis muscle and pelvic floor muscle, including the pelvic muscles in type I and type II, have no statistical difference in two group. The data showed that the incidence of lumbago or dorsalgia in women with DRA and without DRA is almost same.

**Table 2:** The incidence of UI and POP, percentage of weak-RAMs, weak-PFMs and percentage of lumbago or dorsalgia in group of DRA and Non-DRA

	DRA (n = 176)	Non-DRA (n = 37)	*p* value
UI	33 (20.2%)	7 (18.9%)	0.921
POP	13 (7.9%)	3 (8.3%)	0.935
Weak-RAMs	2 (1.2%)	1 (2.7%)	0.502
*Weak-PFMs*			
Weak-PFMs (Type I)	128 (74.9%)	27 (79.4%)	0.572
Weak-PFMs (Type II)	69 (40.6%)	15 (44.1%)	0.703
Lumbago or Dorsalgia	127 (72.6%)	26 (70.3%)	0.777

All of the data above showed as number of cases (valid percentage). DRA, diastasis of rectus abdominis; UI, urinary incontinence; POP, pelvic organ prolapse; RAMs, rectus abdominis muscle strength; PFMs, pelvic floor muscle strength

The association with risk factors for DRA development was described by odds ratio. Postpartum women with history of cesarean section and multiple parturitions are more likely to happen DRA. In keeping with this, the odds ratio for DRA development in our study was 3.48 (95% CI 1.42–8.56), 3.20 (95% CI 1.59–6.45) (Table 3). Physical work and high education were no statistically significant because the 95% confidence interval for the odds ratio contains 1.0.

**Table 3:** Risk factors associated with DRA Development

	OR (95% CI)
Physical work	1.20 (CI 0.60–2.42)
High education	0.74 (CI 0.34–1.58)
Cesarean section	3.48 (CI 1.42–8.56)
Multiple parturition	3.20 (CI 1.59–6.45)

OR, odds ratio; CI, confidence intervals

To study the relationship between the severity of DRA and the occurrence of UI and POP, we use the different cut-off values to determine DRA. The inter-rectus distance is more than 30 mm,40 mm,50 mm separation at any point between the rectus abdominal muscles to be classified the group of DRA-30 mm, DRA-40 mm, DRA-50 mm. As Table 4 showed, no significant difference was found in the prevalence of UI and POP in women with and without diastasis recti abdominis in any of the groups (*p* > 0.05). When the cut-off values of inter-rectus distance more than 30 mm, the proportions of UI and POP in the non-DRA group were higher than the DRA group.

**Table 4:** The incidence of UI and POP in group of different DRA definition

		DRA	Non-DRA	*p* value
DRA-30 mm		102 (47.9%)	111 (52.1%)	
	UI	20 (21.5%)	19 (17.8%)	0.505
	POP	5 (5.2%)	11 (10.7%)	0.150
DRA-40 mm		38 (17.8%)	175 (82.2%)	
	UI	5 (16.7%)	34 (20.0%)	0.671
	POP	0 (0%)	16 (9.7%)	0.055
DRA-50 mm		13 (6.1%)	200 (93.9%)	
	UI	1 (12.5%)	38 (19.8%)	0.610
	POP	0 (0%)	16 (8.6%)	0.272

All of the data above showed as number of cases (valid percentage). DRA, diastasis of rectus abdominis; UI, urinary incontinence; POP, pelvic organ prolapse. DRA-30 mm, DRA-40 mm, DRA-50 mm: DRA was defined as a > 30 mm, 40 mm, 50 mm separation at any point between the rectus abdominis muscles

## Discussion

This study reported that the prevalence of diastasis of rectus abdominis among women during the first postpartum year was 82.6%, which is higher than Sperstad et al. reported as 32.6–60.0% during the same period and the outcome of Boissonnault et al.’s, Mota et al.’s and Gluppe et al.’s [1, 4, 19]. However, the incidence of DRA is variously caused by the different cut-off values, locations between the linea alba and measurements. The reasons why we got this results that we measured the location 4.5 cm above, at and 4.5 cm below the umbilicus, which are different from other studies, and use a distance value greater than 20 mm at any point between the rectus abdominis muscles to define DRA, which is lower than two fingers (almost 30 mm).

The study indicated that there was no statistical difference in relationship of DRA and PFD (including UI and POP). Because the incidence of lumbago or dorsalgia in women with DRA and without DRA is almost the same, conclusion that there was no apparent association between DRA and lumbago or dorsalgia [7, 16, 19] are supported. A systematic review showed that three studies had reported a small association between the DRA and POP, but the methods of these studies were too weak [18]. We used the ultrasound to measure the inter-rectus distance, which is more reliable and objective. This present study is the first report to investigate the correlation between the strength of rectus abdominis muscle, pelvic floor muscle and DRA. Besides, we measured the strength of pelvic floor muscles on different types. It could have more precise conclusion. From the negative results, it can conduct that there is no connection between PFD and DRA.

Risk factors for DRA are controversial. Sperstad et al. [16] reported that age, height, weight gain during pregnancy, caesarean section were not found to be risk factors for happening of DRA [16]. Nevertheless, our study indicated cesarean section and multiple parturitions may be considered as contributing factors to develop the DRA. The proportions of physical work and high education in a group of non-DRA were higher in the DRA group even when there were no statistically significance. But the sample size was smaller than Sperstad et al. [16]. Risk assessment for a condition is useful for prevention and management. The progression of DRA could get better control if we can identify the risk factors in the early period.

A diastasis rectus abdominis of more than 25 mm can be considered harmful due to the influence of the abdominal muscles’ strength [8, 9]. Thus, we analyzed that the postpartum women with the inter-rectus distance of higher than 30 mm, 40 mm or 50 mm at any of the three measurement spots have no statistical significance on the occurrence of UI and POP. It may conclude that there is no association between the occurrence of PFD and the severity of DRA, which is described as the width of inter-rectus distance. Thus, the hypothesis that the severe DRA have higher incidence of PFD is incorrect.

## Strength and limitation

The strengths of our study are (1) this is the first study to determine the sever DRA still have no relationship on the occurrence of PFD; besides when we investigate the correlation of DRA and PFMs, we test PFMs in two types [26, 27]; (2) the measurements of DRA are various which are based on palpation or callipers, ultrasounds; in our study, the measurement for inter-rectus distance is ultrasound, which is more accurate and reliable than fingerbreadth measurement [31, 32]. And we measured three common locations, including above and blew the umbilicus. Even Nicole Beamish et al. found that the inter-rectus distance in women with DRA is not significantly affected by measurement site or task [20]. (3) we asked the same experienced gynaecologist did the ultrasound for inter rectus distance in one room and the same professional physiotherapist performed the assessment of POP-Q, the strength of rectus abdominis muscle and pelvic floor muscle in another room. Base on the above points, we tremendously decrease the subjective bias in this study.

This study has certain limitations. Firstly, this is a retrospective study. The hierarchy of strength of evidence in this type of study is less than the prospective cohort study. Then, sample size plays a significant role in achieving an accurate conclusion. In the stage of study design, we calculated sample size based on the previous study [6, 15], the lowest request sample size is more than 1000 postpartum women for each group. The reason why the request of sample size is too large is that the former researchers have concluded the PFD and DRA were not relative. Even the incidence of PFD in DRA group and non-DRA group in our study is similar with previous studies [6, 15], the participants in our study are so far blew than calculation, which is influenced the conclusion. For method part, even the method of ultrasound is more precise than palpation for measurement of inter-rectus distance, the transducer footprint of ultrasound equipment we used is 60 mm × 18 mm. The size of transducer footprint is bigger than the levels for determining DRA, thus it reduces the accuracy of inter-rectus distance. We measured strength of pelvic muscles and rectus abdominal muscle by manual method testing, which were too subjective. Thus, it should reduce the reliability and validity and increase the bias. However, the levels of PFMs are subjective and depend on the examiner's force-sensing abilities. PFD includes pelvic organ prolapse (POP), urinary incontinence (UI), and sexual dysfunction. The sexual dysfunction is affected by many factors. The data in this study was insufficient for detailed analysis on sexual function. On the other hand, the types of UI were not considered. Thus, the sexual function and the types of UI should be investigated in the further study. Otherwise, in this study we collected all data from the postpartum women, and it could lead to definitely inaccurate results about the relationship between diastasis rectus abdominis and pelvic floor dysfunction. Because the DRA might happen in women who never have a pregnancy.

## Conclusion

In this study, it indicated there were no differences between women with and without DRA groups in the occurrence of UI and POP, even with the severity of inter-rectus distance increasing. Thus, it could say that the DRA did not determine the pelvic floor muscles’ ability and functions. The relationship of the diastasis recti abdominis and pelvic floor dysfunction has no connection. From our findings, the rectus abdominis strength and pelvic floor muscles strength, even for the type I and type II pelvic floor muscles, did not influence the diastasis recti abdominis. Cesarean section and multiple parturitions seem like the more important risk factor for DRA development.

## Data Availability

The datasets generated and analysed during the current study are not publicly available due to privacy protection but are available from the corresponding author on reasonable request.

## Abbreviations

BMI: Body Mass Index
CI: Confidence Intervals
DRA: Diastasis of Rectus Abdominis
IRD: Inter-Rectus Distance
MMT: Manual Muscle Test
OR: Odds Ratio
PFD: Pelvic Floor Dysfunction
PFM: Pelvic Floor Muscles
PFMs: Pelvic Floor Muscles strength
POP: Pelvic Organ Prolapse
POP-Q: Pelvic Organ Prolapse Quantification
RAMs: Rectus Abdominis Muscle strength
UI: Urinary Incontinence
